# Muscle regeneration controlled by a designated DNA dioxygenase

**DOI:** 10.1038/s41419-021-03817-2

**Published:** 2021-05-25

**Authors:** Hongye Wang, Yile Huang, Ming Yu, Yang Yu, Sheng Li, Huating Wang, Hao Sun, Bing Li, Guoliang Xu, Ping Hu

**Affiliations:** 1 State Key Laboratory of Cell Biology, Shanghai Institute of Biochemistry and Cell Biology, Center for Excellence in Molecular Cell Science, Chinese Academy of Sciences, Shanghai, 200031 China; 2 Department of Chemical Pathology, Li Ka Shing Institute of Health Sciences, The Chinese University of Hong Kong, Hong Kong, China; 3 Department of Biochemistry and Molecular Cell Biology, Shanghai Jiao Tong University School of Medicine, Shanghai, 200233 China; 4 Xinhua Hospital affiliated to Shanghai Jiao Tong University School of Medicine, Shanghai, 20023 China; 5 Department of Orthopaedics and Traumatology, Li Ka Shing Institute of Health Sciences, The Chinese University of Hong Kong, Hong Kong, China; 6 State Key Laboratory of Molecular Biology, Shanghai Institute of Biochemistry and Cell Biology, Center for Excellence in Molecular Cell Science, Chinese Academy of Sciences, Shanghai, 200031 China; 7 Key Laboratory of Medical Epigenetics and Metabolism, Institutes of Biomedical Sciences, Medical College of Fudan University, Shanghai, 200032 China; 8 Max-Planck Center for Tissue Stem Cell Research and Regenerative Medicine, Bioland Laboratory (Guangzhou Regenerative Medicine and Health GuangdongLaboratory), Guangzhou, 510005 China; 9 Institute for Stem Cell and Regeneration, Chinese Academy of Sciences, Beijing, 100101 China

**Keywords:** DNA methylation, Muscle stem cells

## Abstract

Tet dioxygenases are responsible for the active DNA demethylation. The functions of Tet proteins in muscle regeneration have not been well characterized. Here we find that Tet2, but not Tet1 and Tet3, is specifically required for muscle regeneration in vivo. Loss of Tet2 leads to severe muscle regeneration defects. Further analysis indicates that Tet2 regulates myoblast differentiation and fusion. Tet2 activates transcription of the key differentiation modulator *Myogenin* (*MyoG*) by actively demethylating its enhancer region. Re-expressing of *MyoG* in Tet2 KO myoblasts rescues the differentiation and fusion defects. Further mechanistic analysis reveals that Tet2 enhances MyoD binding by demethylating the flanking CpG sites of E boxes to facilitate the recruitment of active histone modifications and increase chromatin accessibility and activate its transcription. These findings shed new lights on DNA methylation and pioneer transcription factor activity regulation.

## Introduction

Skeletal muscles can regenerate due to the existence of muscle stem cells (MuSCs)^1,2^. The normally quiescent MuSCs are activated after muscle injury and further differentiate to support muscle regeneration^3,4^. Skeletal muscle development and postnatal muscle regeneration are tightly regulated by muscle-specific transcriptional factors. *MyoD* is considered to be the master regulator of myogenesis^5^, which recognizes and binds E box to activate transcription of target genes^6–10^. Another transcription factors *Myogenin (MyoG)* can also regulate myogenesis^11,12^. MyoD directly activates the transcription of *MyoG* by binding the E-box at its core promoter^8^. Despite the accumulating amount of excellent works about the mechanism of MyoD dependent transcription activation are still needed.

Ten-Eleven Translocation (Tet) family of DNA dioxygenases catalyze the active DNA demethylation and play critical roles in embryonic development, neural regeneration, oncogenesis, aging, and many other important biological processes^13–19^. There are 3 Tet DNA dioxygenase isoforms family in mammal, namely Tet1, Tet2, and Tet3. They share the highly conserved core catalytic domain at the C terminus. During early embryonic development, 3 Tets show functional redundancy^19^. In mammary tissues, Tet2 is predominantly expressed over the other 2 isoforms and promotes luminal lineage commitment^20^. It has been considered that the specificity of Tet2 functions is achieved by predominantly expressing one Tet isoform at a time. In the cases where all Tets are expressed simultaneously at the similar level, whether each Tet has non-redundant functions remains to be explored.

Demethylation of *MyoG* promoter has been shown to contribute to the activation of *MyoG* transcription^21–23^. The functions of Tet2 in vivo during muscle regeneration and its mechanism remain to be further explored.

Here we found that Tet2, but not Tet1 and Tet3, specifically demethylated *MyoG* enhancer. The Tet2 mediated active DNA demethylation at the CpG sites near E boxes enhanced the MyoD binding and increased the chromatin accessibility and active histone modification recruitment. These results revealed the specific functions of Tet2 in vivo during muscle regeneration and shed new lights on understanding the mechanism of transcription regulated by DNA methylation.

## Results

### Tet2 KO mice display muscle regeneration defects

**Fig. 1:**
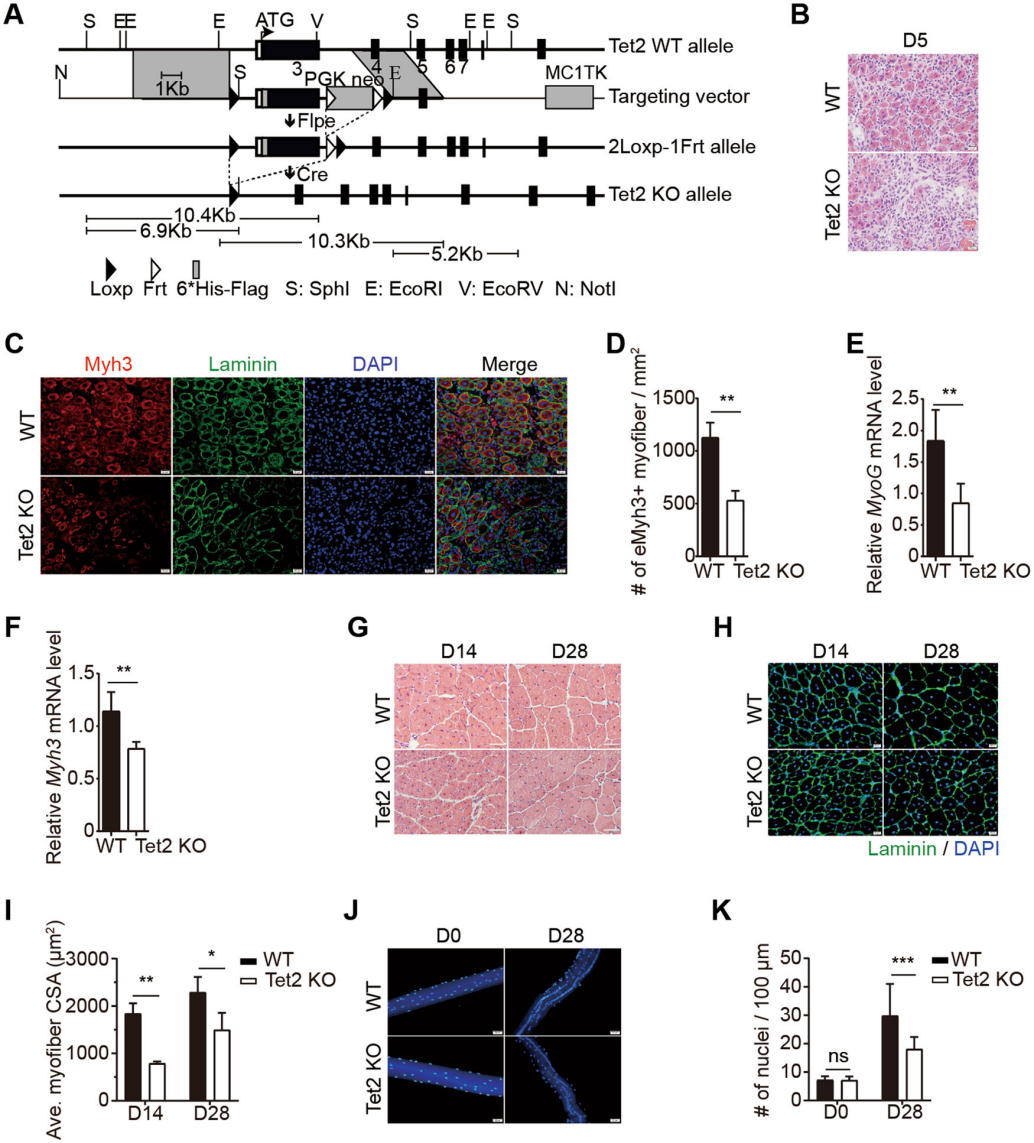
Tet2 KO mice display muscle regeneration defects. **A** The scheme of Tet2 KO strategy. **B** Representative images of H&E staining of TA muscles isolated from WT or Tet2 KO mice on day 5 after injury. Scale bars, 20 μm. **C** Immunofluorescence staining of Myh3 and Laminin of TA muscles isolated from WT or Tet2 KO mice on day 5 after injury. Scale bars, 20 μm. **D** Quantification of the number of Myh3+ myofiber per mm^2^ on day 5 after injury (*n* = 3). **E** Relative mRNA expression level of *MyoG* in TA muscles isolated from WT or Tet2 KO mice on day 3 after injury (*n* = 5). **F** Relative mRNA expression level of *Myh3* on day 5 after injury (*n* = 5). **G** Representative images of H&E staining of TA muscles isolated from WT or Tet2 KO mice on day 14 or day 28 after injury. Scale bars, 50 μm. **H** Immunofluorescence staining of Laminin of TA muscles isolated from WT or Tet2 KO mice on day 14 or day 28 after injury. Scale bars, 20 µm. **I** Quantification of the cross-section area (CSA) from regenerated myofibers on day 14 and 28 after injury (*n* = 3). **J** Representative images of myonuclear staining of myofibers isolated from WT or Tet2 KO TA muscle before injury or on day 28 after injury. Scale bars, 50 µm. **K** Quantification of myonuclei number per 100 µm in single myofiber on day 28 after injury (*n* = 3). *p < 0.05, **p < 0.01, ***p < 0.001.

To identify the function of Tet2 in muscle regeneration, we generated Tet2 knockout mice by deleting exon 3 with homology recombination (Figs. 1A and S1A, B). Tet2 KO mice were born with normal skeletal muscle (Fig. S1C–G), suggesting that Tet2 is not required for muscle embryonic development and postnatal muscle growth.

We then induced muscle injury in Tet2 KO mice by CTX injection. The regeneration defects were observed as indicated by hematoxylin and eosin (H&E) staining and decreased number of Myh3+ newly formed myofibers in Tet2 KO mice (Fig. 1B–D). The expression levels of *MyoG* and *Myh3* were downregulated (Fig. 1E and F). The size of myofibers at the injury site was smaller than that in WT 28 days post injury (Fig. 1G–I) and the number of nuclei in each myofiber also decreased in Tet2 KO mice (Fig. 1J and K).

We then generated Pax7CreERT2: Tet2 flox/flox mice, where Tet2 was knocked out specifically in MuSCs by tamoxifen induction (Fig. S2A). Phenocopying the constitutive Tet2 KO, muscle regeneration defects and smaller myofiber size were observed (Fig. S2B–E). These results suggest that Tet2 KO mice display muscle regeneration defects, especially at the late stage of regeneration.

### Tet2 KO myoblasts display myotube fusion defects

**Fig. 2:**
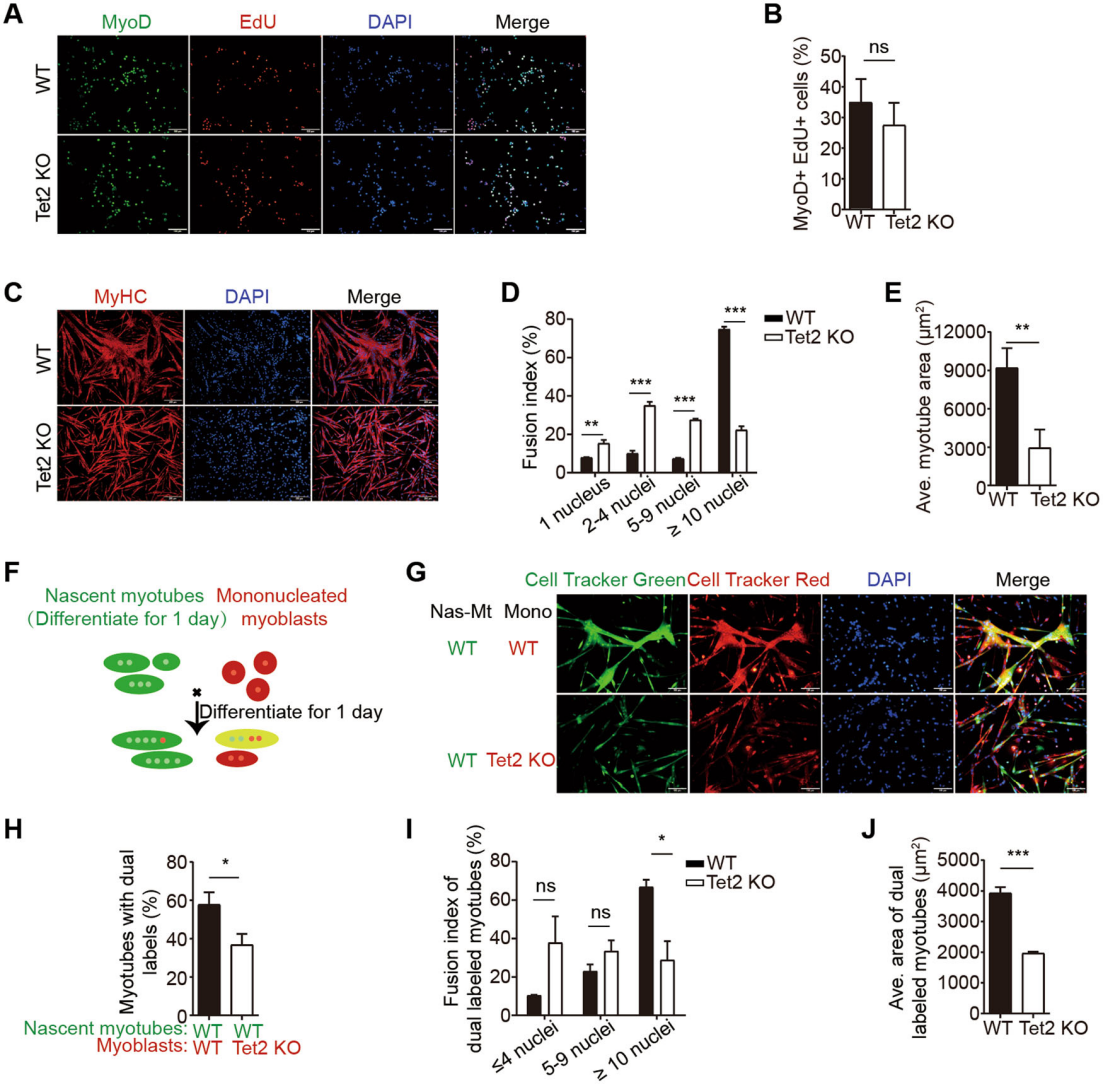
Tet2 KO myoblasts display fusion defects in vitro. **A** Immunofluorescence staining of MyoD and EdU in myoblasts isolated from WT or Tet2 KO mice. Scale bars, 100 µm. **B** Quantification of the percentage of MyoD+ EdU+ myoblasts (*n* = 3). **C** Immunofluorescence staining of MyHC in differentiated myotubes. Scale bars, 200 µm. **D** Quantification of the fusion index in differentiated myotubes (*n* = 3). **E** Quantification of the average area in differentiated myotubes (*n* = 3). **F** Scheme of the second phase fusion assay. **G** Images of the second phase fusion assay results. Scale bars, 100 µm. **H** Quantification of the percentage of the dual labeled myotubes (*n* = 3). **I** Quantification of the fusion index of the dual labeled myotubes (*n* = 3). **J** Quantification of the average areas of dual labeled myotubes (*n* = 3). *p < 0.05, **p < 0.01, ***p < 0.001.

We isolated primary myoblasts from Tet2 KO mice for further examination. The number of MuSCs and the proliferation ability of Tet2 KO myoblasts were not affected both in vitro and in vivo (Fig. 2A, B and Fig. S3A–E). We then checked the differentiation ability of Tet2 KO myoblasts. Tet2 KO myoblasts formed thinner myotubes with lower fusion index (Fig. 2C–E). The expression level of muscle atrophy related genes *MAFbx* (*Atrogin 1*) and *Trim63* (*Murf1*) were unchanged, excluding the possibility of muscle atrophy (Fig. S4A and B) These results together suggest a potential fusion defects of Tet2 KO myoblasts.

To further confirm the fusion defects of Tet2 KO myoblasts, second phase fusion assays were performed as described^24,25^. WT myoblasts were differentiated for 1 day and labeled with Cell Tracker Green. The mononucleated myoblasts isolated from either Tet2 KO or WT mice were labeled with Cell Tracker Red and co-cultured with the green WT myotubes (Figs. 2F and S4C). Both the size and the fusion index of myofibers decreased significantly after co-culturing with Tet2 KO myoblasts (Fig. 2G–J). Taken together, these results suggest that Tet2 KO myoblasts displayed fusion defects.

### Muscle differentiation related genes are downregulated in Tet2 KO myoblasts

**Fig. 3:**
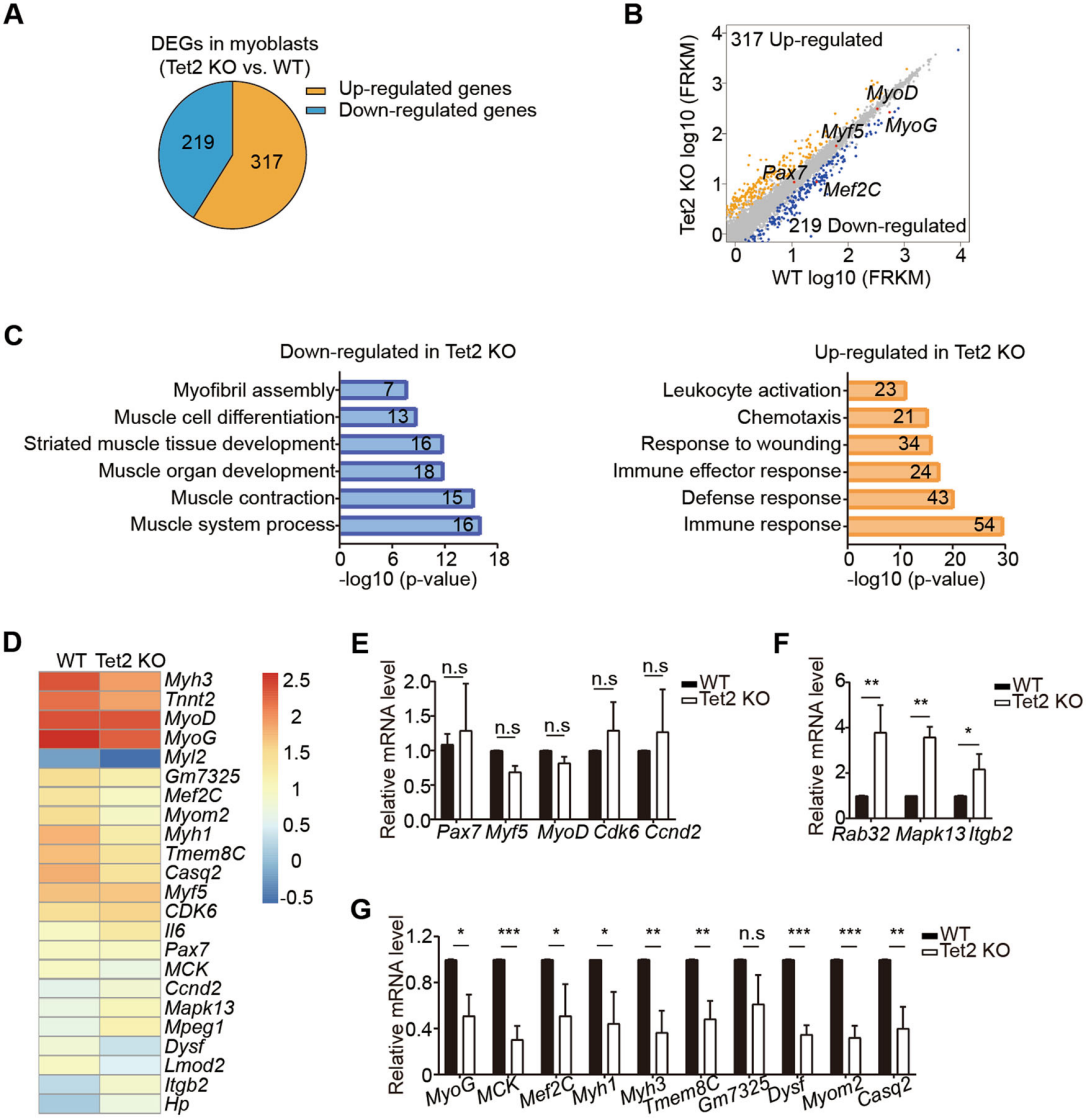
RNA-sequencing analysis of Tet2 KO myoblasts. **A** Pie chart indicated the differentially expressed genes (DEGs) of Tet2 KO myoblasts compared to those of WT myoblasts. **B** Scatter plot indicated the DEGs of Tet2 KO myoblasts compared to those of WT myoblasts. **C** Gene ontology analysis of DEGs in Tet2 KO myoblasts. Numbers within bars indicated the number of genes identified within the corresponding gene ontology categories. **D** Heat map of the expression level of representative genes in myogenesis. **E** Expression levels of the fate determination and proliferation related genes in Tet2 KO myoblasts (*n* = 3). **F** Expression levels of the upregulated genes in Tet2 KO myoblasts (*n* = 3). **G** Expression levels of the differentiation related genes in Tet2 KO myoblasts (*n* = 3). *p < 0.05, **p < 0.01, ***p < 0.001.

To further explore the mechanism, we performed mRNA sequencing using myoblasts isolated from Tet2 KO mice. Consistent with the differentiation defects in Tet2 KO mice, muscle cell differentiation, muscle contraction, and other muscle development related titles were enriched in the downregulated genes (Fig. 3A–C). Consistently, genes related to muscle cell differentiation and myotube fusion were all downregulated (Fig. 3D and G); the expression levels of fate determination and proliferation related genes remained to be unchanged (Fig. 3E); and there were some genes upregulated (Fig. 3F). These results combined suggest that Tet2 regulates the expression of muscle differentiation and myotube fusion related genes.

### Muscle differentiation related genes are hypermethylated in Tet2 KO myoblasts

**Fig. 4:**
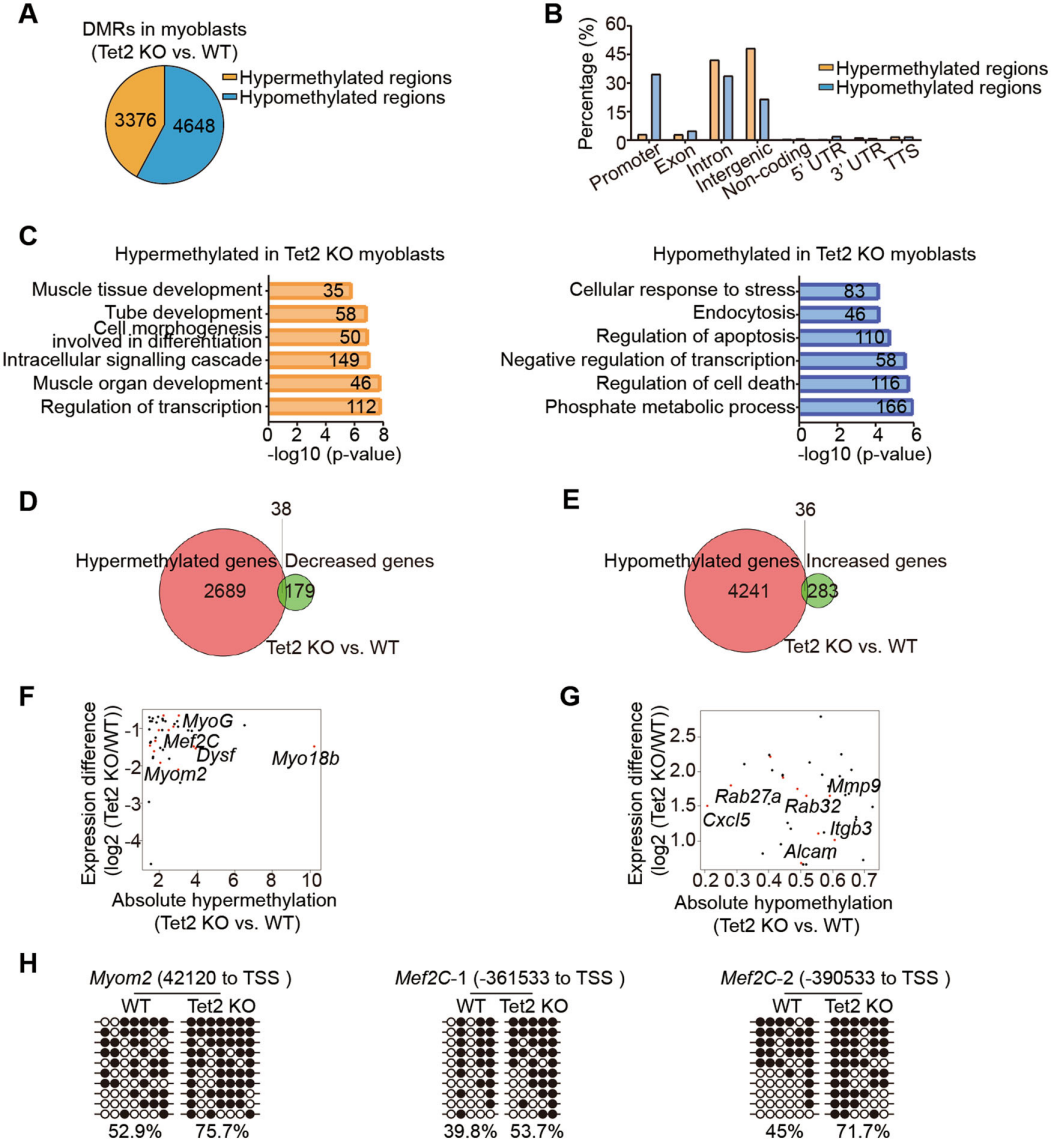
The whole-genome methylation sequencing analysis of Tet2 KO myoblasts. **A** Pie chart indicated the differentially methylated regions (DMRs) of Tet2 KO myoblasts compared to those of WT myoblasts. **B** Distribution of the DMRs at various genomic features. **C** Gene ontology analysis of the DMRs. **D** Venn diagram of the hypermethylated and the downregulated genes in Tet2 KO myoblasts. **E** Venn diagram of the hypomethylated and the upregulated genes in Tet2 KO myoblasts. **F** Scatter plot of the genes hypermethylated and downregulated in Tet2 KO myoblasts. **G** Scatter plot of the genes hypomethylated and upregulated in Tet2 KO myoblasts. **H** The bisulfite sequencing results of *Myom2* and *Mef2C* in WT or Tet2 KO myoblasts. White and black circles indicated hypomethylated and hypermethylated CpG sites, respectively.

Since Tet2 is an mC dioxygenase to remove DNA methylation, we next examined the DNA methylation status in Tet2 KO myoblasts by the whole-genome methylation sequencing. Every 2000 bp in the genome was defined as a region. There were 8024 regions showing DNA methylation change (Fig. 4A). DNA hypermethylation regions were enriched in gene intron and intergenic regions (Fig. 4B), and related to many muscle differentiation associated genes (Fig. 4C).

**Fig. 5:**
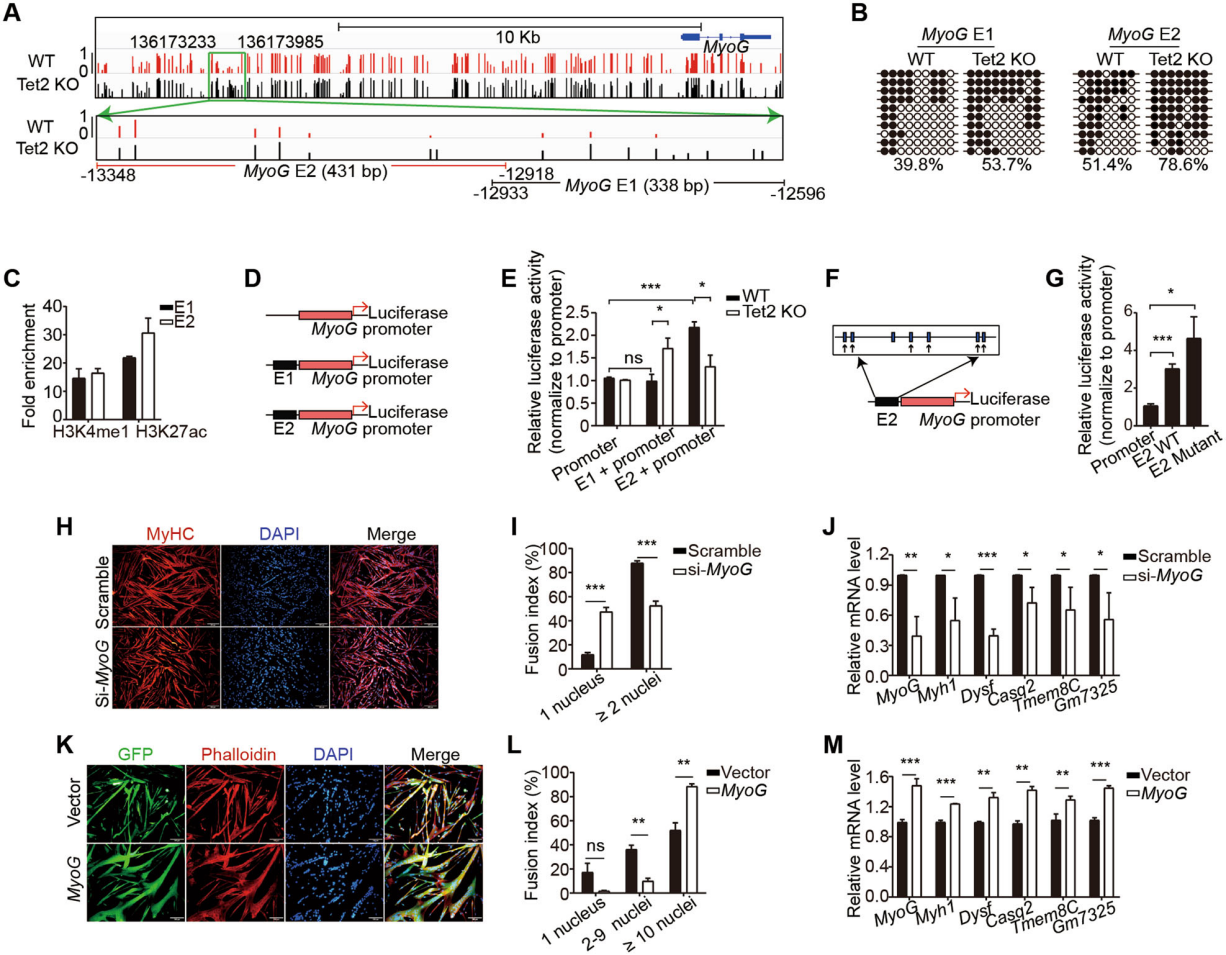
Tet2 targets *MyoG* to facilitate muscle regeneration. **A** Methylation profile of the 5’ upstream region of *MyoG* gene in WT or Tet2 KO myoblasts extracted from genome-wide methylation-seq. **B** Bisulfite sequencing results of E1 and E2 enhancers of *MyoG*. **C** H3K4me1 and H3K27ac levels in E1 and E2 enhancer regions of *MyoG* (*n* = 3). **D** Scheme of the luciferase reporter constructs. **E** Luciferase activity of the E1 or E2 enhancer (*n* = 3). **F** Scheme of the mutated luciferase reporter constructs. Black arrows indicated the mutated CpG sites. **G** Luciferase activity of the E2 enhancer mutant (*n* = 3). **H** Immunofluorescence staining of MyHC in *MyoG* knocked down myotubes. Scale bars, 200 µm. **I** Quantification of the fusion index in *MyoG* knocked down myotubes (n = 3). **J** Relative mRNA expression levels of differentiation related genes in *MyoG* knocked down myotubes (*n* = 3). **K** Immunofluorescence staining of GFP and Phalloidin in *MyoG* over-expressing myotubes. Scale bars, 100 µm. **L** Quantification of the fusion index in *MyoG* over-expressing myotubes (*n* = 3). **M** Relative mRNA expression levels of differentiation related genes in *MyoG* over-expressing myotubes (*n* = 3). *p < 0.05, **p < 0.01, ***p < 0.001.

We further looked for the overlapping genes with decreased expression level and hypermethylation regions in Tet2 KO myoblasts and found 38 genes (Fig. 4D and F). Genes related to muscle differentiation, myotube fusion, and maturation were enriched (Fig. 4F). There were 36 genes with both increased expression levels and hypomethylation (Fig. 4E and G). The bisulfite sequencing assays for the individual gene were performed to confirm the hypermethylation (Figs. 4H and 5B). These results suggest that Tet2 could directly reduce the methylation level of the muscle differentiation related genes and therefore activate their expression.

### Tet2 activates *MyoG* expression by reducing the methylation level of E2 enhancer

*MyoG*, a key myogenic regulator was among the above 38 genes. There was a 753 bp hypermethylation region located −13348 ~ −12596 upstream of *MyoG* transcription starting site (TSS) (Fig. 5A). There were 13 CpG sites within the 753 bp DNA fragment (Fig. 5A). We divided the 753 bp DNA fragment into 2 smaller fragments for further analysis. One 338 bp fragment spanned −12596 ~ −12933 bp region was named E1; the other 431 bp fragment spanned −12918 ~ −13348 bp region was named E2 (Fig. 5A). Both E1 and E2 were hypermethylated in Tet2 KO myoblasts (Fig. 5B). Chromatin immunoprecipitation (ChIP) assays were performed to survey the histone marks marking the active enhancer. H3K4me1 and H3K27ac were enriched at both E1 and E2 (Fig. 5C), suggesting them to be enhancers for *MyoG*. E1 or E2 was inserted directly upstream of the *MyoG* promoter to drive the expression of luciferase (Fig. 5D). The constructs were transfected to the primary myoblasts isolated from WT or Tet2 KO mice. In the WT myoblasts, E2 enhancer activated *MyoG* transcription, while E1 enhancer barely worked (Fig. 5E). When the same construct was transfected to the primary Tet2 KO myoblasts, the activation of E2 enhancer was diminished (Fig. 5E). The Cs in E2 enhancer were randomly mutated to non-C nucleotides to abolish its ability to be methylated (Fig. 5F). The non-methylation mutant E2 enhancer showed higher transcription activation ability (Fig. 5G). These results together suggest that methylation of E2 enhancer by Tet2 down-regulates *MyoG* transcription activity.

To confirm that *MyoG* is the major target of Tet2, *MyoG* expression was knocked down in WT primary myoblasts (Fig. S5A and B). Similar to Tet2 KO myoblasts, the fusion index of *MyoG* knocked-down cells decreased after differentiation (Fig. 5H and I). The expression of the similar set of genes was downregulated both in Tet2 KO cells and in *MyoG* knocked-down cells (Fig. 5J). When *MyoG* was re-expressed in Tet2 KO myoblasts (Fig. S5C), the fusion defect and the expression of the fusion related genes were rescued (Fig. 5K–M)), suggesting that *MyoG* is the key target of Tet2 in muscle cells.

### Tet2 mediated DNA demethylation increases MyoD binding affinity at the neighboring E boxes

**Fig. 6:**
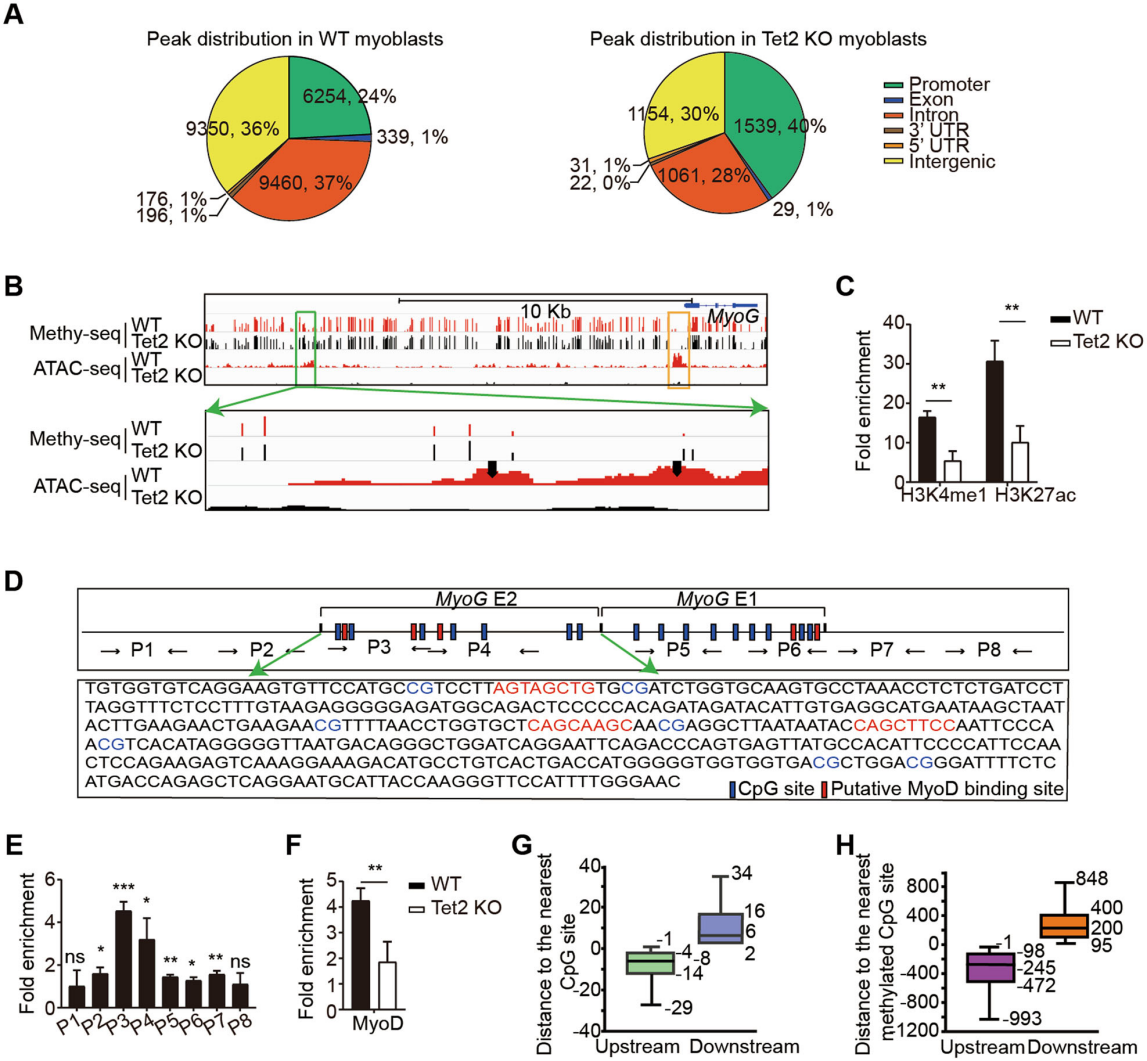
Tet2 regulates chromatin accessibility and MyoD binding in the E2 enhancer of *MyoG*. **A** Pie chart of ATAC-seq analysis. **B** Methylation and chromatin accessibility profile of the 5′ upstream region of *MyoG* gene in WT or Tet2 KO myoblasts extracted from the genome-wide methylation-seq and ATAC-seq. The black arrows indicated the major chromatin accessibility peaks absent in Tet2 KO myoblasts. **C** The H3K4me1 and H3K27ac levels on E2 enhancer of *MyoG* gene in Tet2 KO myoblasts (*n* = 3). **D** Structure of the *MyoG* enhancer region. **E** MyoD binding profile in *MyoG* enhancer region (*n* = 3). **F** The level of MyoD recruitment on E2 enhancer in Tet2 KO myoblasts (*n* = 3). **G** The distance of CpG sites to E boxes bound by MyoD regardless of methylation status. **H** The distance of methylated CpG sites to E boxes bound by MyoD. *p < 0.05, **p < 0.01, ***p < 0.001.

We next set out further mechanism exploration. ATAC-sequencing analysis was performed with myoblasts isolated from Tet2 KO or WT mice. The number of peaks decreased over 6 folds in Tet2 KO myoblasts, suggesting a significant decrease of chromatin accessibility with the absence of Tet2. The distribution of peaks was also changed in Tet2 KO myoblasts, especially at the enhancer region (Fig. 6A), suggesting the Tet2 dependent chromatin accessibility at the enhancer region. Consistently, the genes related to muscle cell differentiation also showed reduced chromatin accessibility in Tet2 KO myoblasts (Fig. S6A). The chromatin accessibility decreased dramatically around the E2 enhancer hypermethylation region (Fig. 6B). In addition to the general decrease of chromatin accessibility in Tet2 KO myoblasts (Fig. S6B), some major peaks around the hypermethylated CpG sites were almost completely lost in E2 enhancer in Tet2 KO myoblasts (Fig. 6B, black arrows), suggesting that the chromatin accessibility at E2 enhancer is more sensitive to DNA hypermethylation. Furthermore, the levels of H3K4me1 and H3K27ac were downregulated at E2 enhancer in Tet2 KO myoblasts (Fig. 6C). Taken together, these results suggest that the increased DNA methylation at the enhancer region leads to reduced chromatin accessibility, decreased level of active histone marks, and declined transcription activity of *MyoG*.

*MyoD* is the key transcription factor activating the transcription of *MyoG* and inducing chromatin accessibility changes^12,26^. We then investigated the link between MyoD binding and Tet2. By sequencing analysis, we found 3 putative MyoD recognition sites at E2 enhancer (Fig. 6D). Interestingly, CpG sites were found within 10 bp of the putative MyoD recognition sites (Fig. 6D). The recruitment of MyoD on E2 enhancer was surveyed by ChIP assays with a series of primers spanning the enhancer region (Fig. 6E). The binding of MyoD at E2 enhancer was reduced in Tet2 KO myoblasts (Fig. 6F), suggesting that the hypermethylation at the neighboring CpG sites represses MyoD binding.

We then performed bioinformatic analysis to identify the pattern of the methylation at the flanking CpG sites and MyoD binding in the whole-genome. By analyzing the MyoD ChIP-seq data in myoblasts collected in both ENCODE and GEO (GEO number: GSM915186), we found that CpG sites, regardless of the methylation status, were located about 6–8 bp upstream or downstream of E boxes bound by MyoD (Fig. 6G). In sharp contrast, the methylated CpG sites were located 200–245 bp away from the E boxes bound by MyoD (Fig. 6H), suggesting the loss of MyoD binding on sites with nearby methylated CpG sites.

We further surveyed the consensus sequence of E boxes in WT and Tet2 KO myoblasts. The top consensus sequence of MyoD in WT and Tet2 KO hypermethylation regions was the canonical CAGCTG sequence (Fig. S7). Some of the less prominent consensus sequences were absent in the hypermethylated regions, such as GGGAAR, CACACA, and AKAAAH (Fig. S7), suggesting that MyoD binding on these motifs are repressed by the neighboring CpG methylation. In contrast, some motifs like TTTAWW, CTGTGK, and CAGRTG were only present in the hypermethylated region, suggesting that the methylation of the flanking CpG sites improve MyoD binding. These results together suggest that the binding affinity of MyoD on E boxes is regulated by the methylation level of the neighboring CpG sites in a sequence dependent manner.

### Tet2 specifically promotes myoblast differentiation

**Fig. 7:**
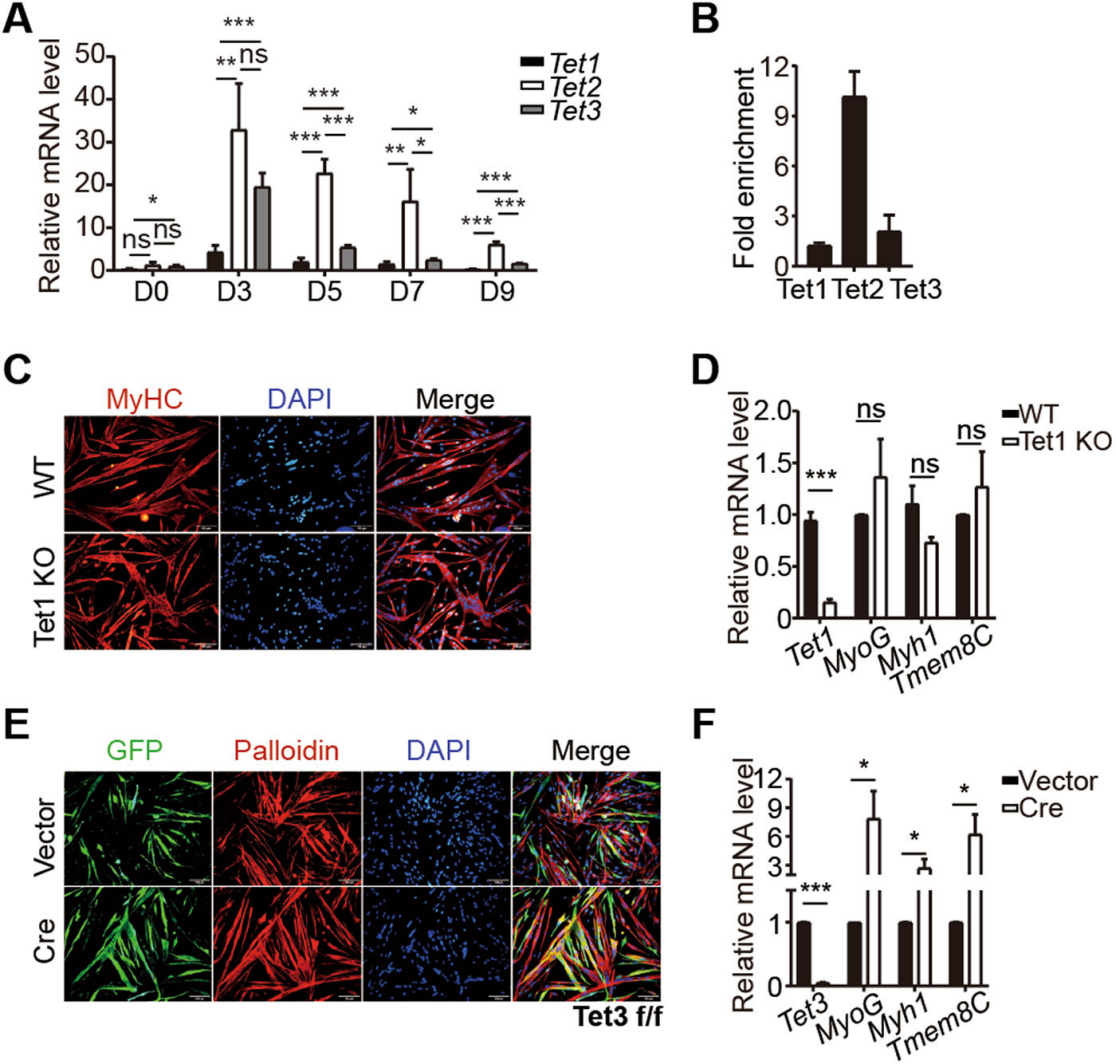
Tet2, but not Tet1 and Tet3, specifically promotes myogenesis. **A** The relative expression levels of *Tet1*, *Tet2*, and *Tet3* during muscle regeneration (*n* = 3). **B** Tet2 recruitment on E2 enhancer (*n* = 3). Myoblasts were isolated from Tet1-HA, Tet2-HA, Tet3-HA mice, respectively. ChIP-qPCR assays were performed using anti-HA. **C** Immunofluorescence staining of MyHC in Tet1 KO myotubes. Scale bars, 100 µm. **D** Relative mRNA expression level of *Tet1*, *MyoG*, *Myh1*, *Tmem8C* in Tet1 KO myoblasts (*n* = 3). **E** Immunofluorescence staining of GFP and Phalloidin in Tet3 KO myotubes. Scale bars, 100 µm. **F** Relative mRNA expression level of *Tet3*, *MyoG*, *Myh1*, *Tmem8C* in Tet3 KO myoblasts (*n* = 3). *p < 0.05, **p < 0.01, ***p < 0.001.

All 3 isoforms of Tet dioxygenases were upregulated during muscle regeneration process (Fig. 7A). We then explored whether the promotion of myogenesis is a common function of all Tet dioxygenases. ChIP assays were performed in myoblast to survey the binding of Tet1, Tet2, and Tet3 on E2. To avoid the variation caused by different antibodies, HA tag was knocked in at the C terminus of Tet1, Tet2, or Tet3 mice, and the primary myoblasts were isolated. Surprisingly, only Tet2, but not Tet1 and Tet3, was enriched at the E2 enhancer (Fig. 7B), suggesting that Tet2 is specifically recruited to the E2 enhancer.

Differentiation ability of myoblasts isolated from Tet1 KO mice was checked. In contrast to Tet2 KO, these cells differentiated normally and the expression of differentiation related genes remained unchanged (Fig. 7C and D). Tet3 KO mice are embryonic lethal^27^. We isolated myoblasts from Tet3 flox/flox mice and infected them with adenovirus encoding Cre recombinases to knock out Tet3 (Fig. 7E). Unexpected, the Tet3 KO myoblasts showed enhanced differentiation ability and the differentiation related genes were upregulated (Fig. 7E and F). These results suggest that Tet2 regulates *MyoG* transcription specifically and each Tet enzyme plays unique roles during skeletal muscle regeneration.

## Discussion

The functions of the active demethylation in muscle regeneration in vivo have not been fully characterized. Here we report that Tet2 DNA dioxygenase, but not Tet1 and Tet3, specifically demethylates the CpG sites at the close approximation to E boxes to enhance MyoD binding and further facilitates the chromatin accessibility and recruitment of active histone modifications at the *MyoG* enhancer to regulate muscle regeneration.

The transcription activity of *MyoG* has been implicated to show close ties with the DNA methylation level^21,23^. Tet2 has been shown to play an important role in regulating C2C12 differentiation^23,28,29^. Here we further show that Tet2, but not Tet1 and Tet3, specifically binds *MyoG* enhancer to activate *MyoG* transcription, further emphasizing the importance of Tet2 in myogenesis. Compared to Tet1 and Tet3, Tet2 lacks the CXXC domain^17^. CXXC domain has been suggested to direct Tet binding specificity^30,31^. The CXXC domain interaction achieved by protein-protein interactions between Tets and other proteins may attribute to the specific binding of each individual Tet isoform.

*MyoD* is considered to be the “master transcription factor” and a pioneer factor to determine the muscle lineage^6,7,32^. ChIP-seq results reveal that there are thousands of constitutive MyoD binding irrelevant to transcription activation and muscle differentiation^33^. The specific activation of differentiation related genes by MyoD may require further regulation. Here we found that the demethylation of the flanking CpG sites within 100 bp to E boxes increases the MyoD binding affinity. Our findings suggest that the methylation of the flanking region of the DNA elements is also the key element to regulate transcription factor binding affinity.

## Materials and methods

### Animals

Housing, mating and all experimental protocols for mice used in this study were performed in accordance with the guidelines established by the Institutional Animal Care and Use Committee in Shanghai Institute of Biochemistry and Cell Biology, Chinese Academy of Sciences. C57BL/6 were obtained from SLRC Laboratory Animal. Tet2 KO, Tet2 flox/flox, Tet1 KO, Tet3 flox/flox, Tet3-HA mice were kindly provided by Dr. Guoliang Xu (Shanghai Institute of Biochemistry and Cell Biology, CAS). Tet1-HA and Tet2-HA tagged mice was kindly provided by Dr. Jingsong Li (Shanghai Institute of Biochemistry and Cell Biology, CAS). Pax7 CreERT2 mice were purchased from Jackson Laboratory (JAX, stock #017763). Tet2 flox/flox mice were crossed with Pax7 CreERT2 mice to generate Pax7 CreERT2; Tet2 flox/flox mice. If not stated differently, 8–10-week-old male mice were used for all experiments.

Conditional knockout was induced by tamoxifen (Sigma) injection as described previously^34^. In brief, 10 mg/ml tamoxifen (Sigma) suspended in corn oil (Sigma) was injected intraperitoneally into 6-week-old Pax7-CreERT2: Tet2 flox/flox mice for 5 consecutive days at a dose of 100 mg/kg body weight per day. Littermates of the same genotype were injected with corn oil as a vehicle control.

### Antibodies

Antibodies used for flow cytometry were AF700-anti-mouse Sca-1 (Thermo, 56-5981-82), PerCP/Cy5.5-anti-mouse CD11b (BD Biosciences, 550933), PerCP/Cy5.5-anti-mouse CD31 (BD Biosciences, 562861), PerCP/Cy5.5-anti-mouse CD45 (BD Biosciences, 550944), FITC anti-mouse CD34 (BD Biosciences, 553733), APC-anti-Integrin a7+ (R&D, FAB3518A).

Antibodies used for western blots were Myogenin (F50D) (Santa Cruz, SC-12732), Flag-Tag (3B9) mAb (Abmart, M20008), GAPDH (14C10) Rabbit mAb (Cell Signaling, #2118), Goat anti-mouse IgG-HRP (Santa Cruz, SC-2005), Goat anti-rabbit IgG-HRP (Santa Cruz, SC-2004).

Antibodies used for immunofluorescence staining were anti-MyoD (Santa Cruz, sc-377460), anti-Pax7 (DHSB, RRID:AB_528428), anti-Myh3 (DHSB, F1.625), anti-MyHC (Millipore, 05-716), anti-Laminin (Abcam, ab11575), anti-GFP (Aves Labs, GFP-1010).

Antibodies used for ChIP assays were anti-H3K4me1 (Abcam, ab8895), anti-H3K27ac (Abcam, ab4729), HA antibody (generated by our lab), mouse IgG (Abmart, B30010M), rabbit IgG (Abmart, B30011M).

### Cardiotoxin (CTX) injection

Muscle injury was induced by intramuscular injections of CTX (Sigma-Aldrich, C3987) into TA muscle as previously described)^34,35^. Each mouse was injected with 100 μl 10 μM CTX using 28-gauge needle at multiple injection sites in TA muscle. Mice were put under suction anesthesia with isoflurane during injection.

### C2C12 cell culture

C2C12 cells were cultured at 37 °C with 5% CO_2_ in Dulbecco’s modified eagle’s medium (DMEM, Invitrogen) supplemented with 10% fetal bovine serum (FBS, Hyclone) and 100U/ml penicillin/streptomycin (Invitrogen, 15140-122).

### Primary myoblasts isolation, expansion, and differentiation

Primary myoblasts were isolated as previously described^35^. Briefly, dissected TA muscles were digested with 10 ml muscle digestion buffer (DMEM containing 1% penicillin/streptomycin, 0.125 mg/ml Dispase II (Roche, 04942078001), and 10 mg/ml Collagenase D (Roche, 11088866001)) for 90 min at 37 °C. The digestion was stopped by adding 2 ml of FBS. The digested cells were filtered through 70 μm strainers. Red blood cells were lysed by 7 ml RBC lysis buffer (0.802% NH_4_Cl, 0.084% NaHCO_3_, 0.037% EDTA in ddH_2_O, pH7.2–7.4) for 30 s, then filter through 40 μm strainers. After staining with antibody cocktails (AF700-anti-mouse Sca-1, PerCP/Cy5.5-anti-mouse CD11b, PerCP/Cy5.5-anti-mouse CD31, PerCP/Cy5.5-anti-mouse CD45, FITC anti-mouse CD34, APC-anti-mIntegrin a7+), the mononuclear cells were subjected for FACS analysis using Influx (BD Biosciences). The population of PI-CD45-CD11b-CD31-Sca1- CD34+ Integrin a7+ cells was collected.

Primary myoblasts were cultured on 0.5 mg/ml Type I collagen (Corning, 354249) coated dish as described previously^35^ and differentiated in differentiation medium (DMEM containing 2% horse serum (Hyclone, SH30074.03) and 100 μ/ml penicillin/streptomycin (Hyclone, SV30010)) for 48 h.

### Myofiber isolation

Myofibers were isolated as described previously^36^. Briefly, TA muscles (uninjured and injured for 28 days) were isolated from tendon to tendon. The isolated TA was digested in DMEM medium (Gibco, 11965118) contain 0.2% collagenase D (Roche, 11088866001) for 90 min to disassociate myofibers. The digested muscle tissues were triturate three times with a wide bore 5 ml pipet. Sit at room temperature for 5 min to allow fibers to settle down. Remove the supernatant and wash the myofibers with pre-warmed DMEM medium twice. Pick out single fibers with wide bore yellow tips and transfer directly to 4% paraformaldehyde to fix for 20 min.

### Immunofluorescence staining

TA muscle samples were embedded in OCT (Thermo Fisher Scientific, 6506), and frozen in liquid nitrogen for 20 s then cut for 8–10 μm thick cryosections. Cultured cells or cryosections were fixed in 4% paraformaldehyde for 20 min at room temperature and washed 3 times with PBS. Samples were permeabilized with 0.5% Triton X-100 for 15 min, and blocked in 3% goat serum (Gibico, 16210072) for 60 min at room temperature. The samples were incubated in primary antibody for overnight followed by 3 washes with PBS. They were then incubated in fluorescent labeled secondary antibody followed by 3 washes with PBS and DAPI(0.5 μg/ml) (Invitrogen, D3571) staining. The mounted slides were visualized by BX53 microscope (Olympus).

Pax7 immunofluorescent staining was carried out as described previously^34,35^. Briefly, the cryosections were permeabilized with pre-chilled methanol for 6 min at −20 °C and blocked with M.O.M. Blocking Reagent (Vector, MKB-2213) for 2 h. The primary and secondary antibody incubation steps were the same as described above.

### EdU labeling

Primary myoblasts were labeled with 10 μM EdU for 2 h at 37 °C followed by fixation with 4% paraformaldehyde, permeabilized with 0.5% Triton X-100, then reacted with Click-iT reaction cocktail (Invitrogen, C10338) and counterstained with Hoechst 33342 (Sigma, B2261). 50 μg/g body weight EdU (Sigma, 900584) was injected intraperitoneally for two consecutive days before sacrifice. EdU staining was performed with Click-iT EdU Cell Proliferation Assay kit (Invitrogen, C10338).

### Cell fusion experiment

Cell fusion was measured as previously described^24,25^. MuSCs were differentiated for 24 h in differentiation medium (DMEM containing 2% horse serum and 100 μ/ml penicillin/streptomycin). The nascent myotubes were stained with 0.5 μM Cell Tracker™ Green CMFDA Dye (Invitrogen, C2925) for 10 min at 37 °C. The mononucleated myoblasts were stained with 0.5 μM Cell Tracker™ Red CMTPX Dye (Invitrogen, C34552) for 10 min at 37 °C and added to the primary myotubes to further differentiate for another 24 h. The cells were fixed with 4% paraformaldehyde and visualized by BX53 microscope (Olympus).

### ChIP assay

ChIP assays were performed as previously described^37,38^. Briefly, crosslinking was performed in 1% formaldehyde for 10 min at room temperature, then stopped by 125 mM glycine for 5 min. Nuclei were isolated in cell lysis buffer (50 mM pH7.6 HEPEs, 10 mM KCl, 1.5 mM MgCl_2_, 1 mM EDTA, 0.5 mM EGTA, 0.5% Triton X-100, add protease inhibitors (1 mM PMSF, 1 mM DTT) freshly) and chromatin was further extracted from the nuclei using nuclei lysis buffer (50 mM pH7.6 HEPEs, 1 mM EDTA, 0.5 mM EGTA, 1% Triton X-100, 0.1% deoxycholate, add protease inhibitors freshly). The chromatin was then sheared to 200–500 bp by sonication (Bioruptor) and applied for antibody precipitation. Dynabeads^TM^ Protein G (Invitrogen, 10004D) were used to capture the precipitated chromatin by antibody and followed by washes and reverse crosslinking. The purified DNA fragments were detected by qPCR. The fold enrichment were calculated against IgG ChIP-qPCR. Primers were listed in Table S1.

### RT-qPCR

Total RNA was extracted by TRIzol Reagent (Thermo, 15596-018), and reverse transcription was performed using reverse-transcriptase M-MuLV (NEB, M0253L) to generate cDNA, followed by. qPCR analysis using SYRB Green qPCR Mix (Roche, A0001) by QuantStudio6 Flex (Thermo). The expression level of each gene was normalized to that of GAPDH. Primers were listed in Table S2.

### Measurement of myofiber size and fusion index

The myofiber cross-section area was measured by Image Pro Plus software. At least 100 myofibers were measured for each sample. The number of regenerated myofibers and the fusion index (the number of nuclei in differentiated myotubes) were also counted by Image Pro Plus software. At least 3 fields were measured for each sample. The identity of the samples was blinded to the personnel who performed the measurement.

### Statistical analysis

The numbers of biological replicates and technical repeats in each experimental group were indicated in figure legends. For single myofiber staining, at least 10 single myofibers were isolated from each mouse. For myofiber CSA quantification, at least 100 myofibers from each mouse was quantified. For myoblasts proliferation and differentiation quantification, at least 3 microscope field were counted for each sample. Error bars represented standard deviation unless noted otherwise. Statistical differences between groups were determined by unpaired two-tailed *t*-test in GraphPad Prism 8 software. Statistical significance was set at *p* < 0.05. ns indicated no significant difference, * indicated *p* < 0.05, ** indicated *p* < 0.01, *** indicated *p* < 0.001.

### RNA interference

C2C12 myoblasts or primary myoblasts were transfected by siRNAs using Lipofectamine™ LTX reagent (Invitrogen, 15338030) following the manufacturer’s instructions. At least 2 pieces of siRNA were used for each target gene.

si*MyoG* sense-1 (5′–3′): GCAUCACGGUGGAGGAUAUTT;

si*MyoG* antisense-1 (5′–3′): AUAUCCUCCACCGUGAUGCTT;

si*MyoG* sense-2 (5′–3′): GCAUGUAAGGUGUGUAAGATT;

si*MyoG* antisense-1 (5′–3′): UCUUACACACCUUACAUGCTT;

siControl sense (5′–3′): UUCUCCGAACGUGUCACGUTT;

siControl antisense (5′–3′): ACGUGACACGUUCGGAGAATT.

### Luciferase reporter assay

*MyoG* promoter (+3 ~ −1596), *MyoG* promoter + E1 (WT or Mutation), and *MyoG* promoter + E2 (WT or Mutation) were cloned into pGL3 basic vector and transfected to primary myoblasts using the Neon™ Transfection System Starter Pack (Invitrogen, MPK5000S), respectively. After transfection 48 h, luciferase activities were measured using Dual-Luciferase Reporter Assay System (Promega, PR-E1910) by BioTek Synergy NEO (BioTek) following the manufacturer’s instructions. Relative luciferase activity was calculated as the ratio of Firefly/Renilla luciferase activity. All experiments were repeated at least 3 times. Primers were listed in Tables S3 and S4.

### Conventional bisulfite sequencing

Genomic DNA was extracted and treated with EZ DNA Methylation-Direct Kit (Zymo Research, D5005) according to the manufacturer’s instructions. Bisulfite-treated DNA was subjected to PCR amplification, then purified with the Gel Extraction Kit (TianGen, DP204) and cloned into pGEM-T vector system I (Promega, A3600). Individual clones were sequenced by standard Sanger sequencing. Data were analyzed by BISMA (http://services.ibc.uni-stuttgart.de/BDPC/BISMA/)^39^. Primers were listed in Table S5.

### RNA-seq

Total RNA was extracted by TRIzol Reagent (Thermo, cat# 15596-018) to constructed library using TruSeq RNA Library Preparation Kit v2 (Illumina, RS-122-2001). The raw RNA-seq reads were first evaluated by FastQC^40^ and preprocessed by Illumina universal adapter trimming and low-quality reads filtering with our in-house scripts^41^. All clean paired-end reads were then mapped to the mouse genome (mm9) using Tophat^42^ (version 2.0.13). Following alignment and filtering, gene expression levels were estimated by counting the number of fragments that are mapped to per kilobase of transcript per million mapped reads (FPKM). Differentially expressed genes (DEGs) between every pairs of KO and WT samples were analyzed with Cuffdiff^43^ (version 2.2.1) and genes with an adjusted *p*-value (*q*-value) <0.05 were identified as DEGs.

### Whole-genome methylation-seq

Genomic DNA was extracted and 1 μg genomic DNA was treated with EZ DNA Methylation-Gold Kit (Zymo Research, D5005) according to the manufacturer’s instructions. The methylation-seq library was constructed using TruSeq Nano DNA High Throughput Library Prep Kit (Illumina, 20015965). The bisulfite-treated DNA methylation sequencing data were analyzed by a data analysis pipeline called Methy-Pipe^44^. The paired-end reads were first aligned to the reference mouse genome (mm9) by the BSAligner module. The sequencing adaptors and low-quality bases on read ends were trimmed. All Cs in both the reference genome and sequenced reads were replaced by Ts in silico and the pre-processed and converted reads were aligned to the converted reference genome. Then methylation density (MD) calculation and the DMRs identification were performed by BSAnalyzer module. After MD calculation, a sliding window approach is employed and Mann–Whitney *U* test is recruited to identify differentially methylated seed regions (*p*-value <0.01). Regions with at least 20% changes of absolute methylation level and *p* < 0.01 were defined as DMRs. Finally, we defined the merged seed regions with significant difference as putative DMRs.

### ATAC-seq

ATAC-seq library was constructed from 5000 cells using TruePre DNA Library Pre Kit V2 for Illumina (Vazyme, TD501-01) and big peaks were filtered out using VAHTS DNA Clean Beads (Vazyme, N411-02) according to the manufacturer’s instructions. These libraries were sequenced on Illumina HiSeq X Ten instrument with 150-bp reads and paired-end parameter by WuXi NextCODE company. The 150 bp paired-end ATAC-Seq reads were trimmed to 36 bp paired-end reads and aligned to the mouse reference genome (mm9) using Bowtie2^45^ with parameters -X 2000 -no-mixed -no-discordant -local. Aligned reads are filtered for mapping quality ≥30 and duplicate reads was removed by using the Picard MarkDuplicates (https://www.broadinstitutegithubio/picard/). Peak regions of each sample were called by software MACS2^46^ with options -f BAMPE -g mm -q 0.01. For visualization, the alignment bam files were converted into bedgraph files using homer (http://homer.ucsd.edu/homer/ngs/ucsc.html). Differential peaks were adjusted by DESeq2 with fold change >2 and *p* < 0.05.

## Supplementary information

Fig. S1

Fig. S2

Fig. S3

Fig. S4

Fig. S5

Fig. S6

Fig. S7

Table S1

Table S2

Table S3

Table S4

Table S5

## Data Availability

All the sequencing data (mRNA-seq, methylation-seq, ATAC-seq) in WT and Tet2 KO primary myoblasts are available through GEO under the accession number GEO: GSE158649.
