# Supplementary figures and images for: Muscle regeneration controlled by a designated DNA dioxygenase
Source: Cell Death Dis. 2021 May 25;12(6):535. doi: 10.1038/s41419-021-03817-2 (PMC8149877; doi:10.1038/s41419-021-03817-2)

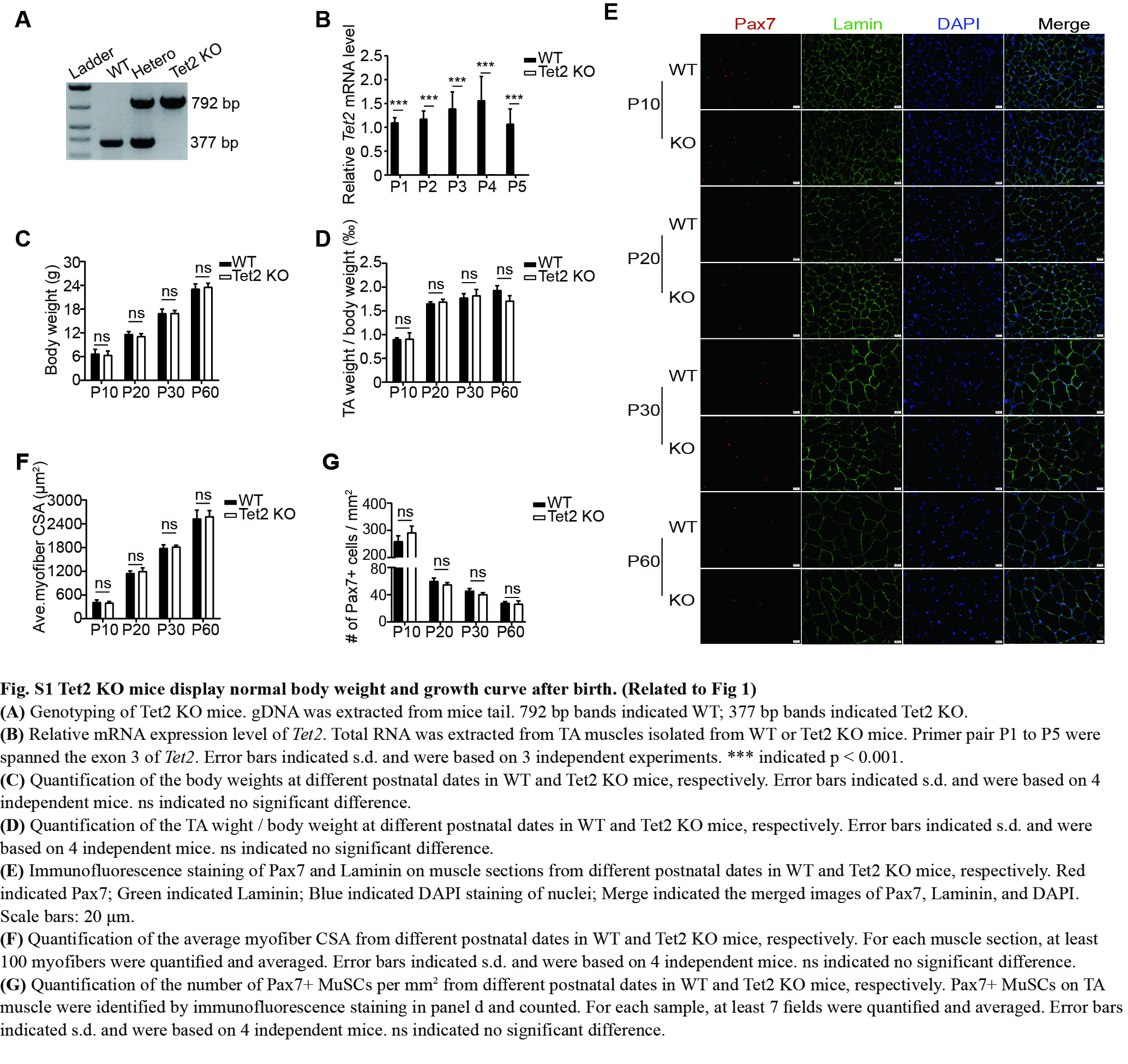

Supplement: Supplementary file 1 — Fig. S1 [file 41419_2021_3817_MOESM1_ESM.tif]

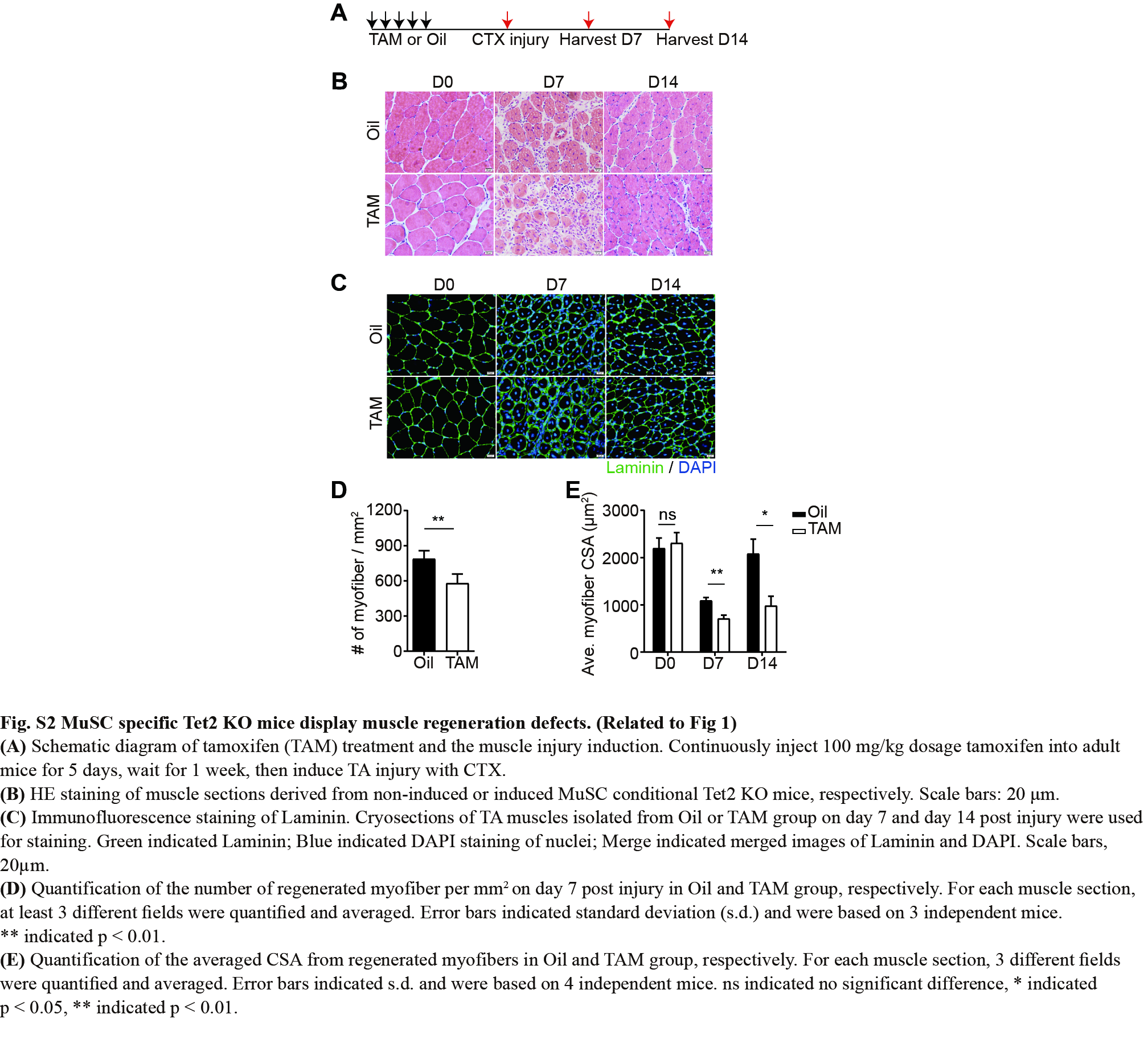

Supplement: Supplementary file 2 — Fig. S2 [file 41419_2021_3817_MOESM2_ESM.tif]

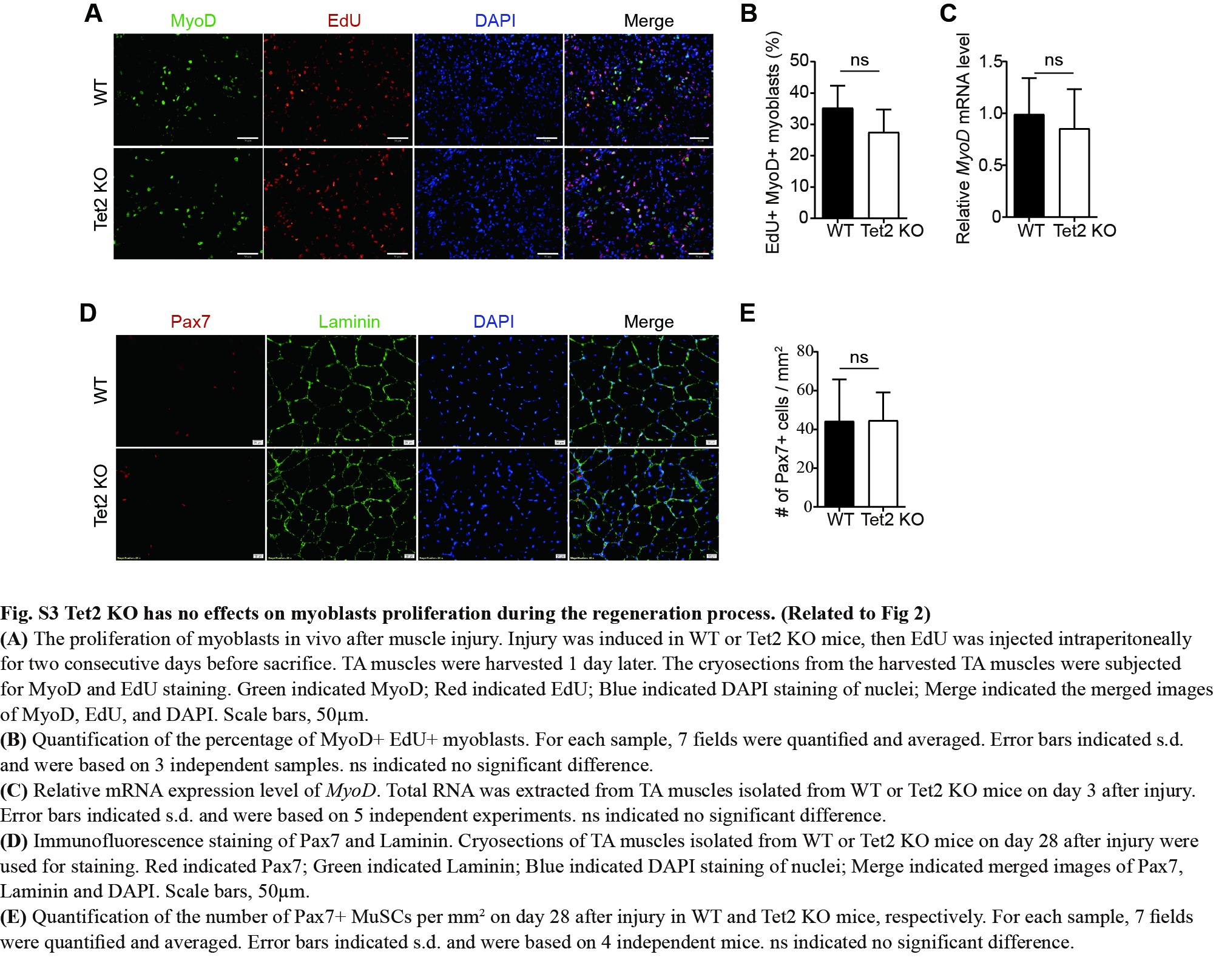

Supplement: Supplementary file 3 — Fig. S3 [file 41419_2021_3817_MOESM3_ESM.tif]

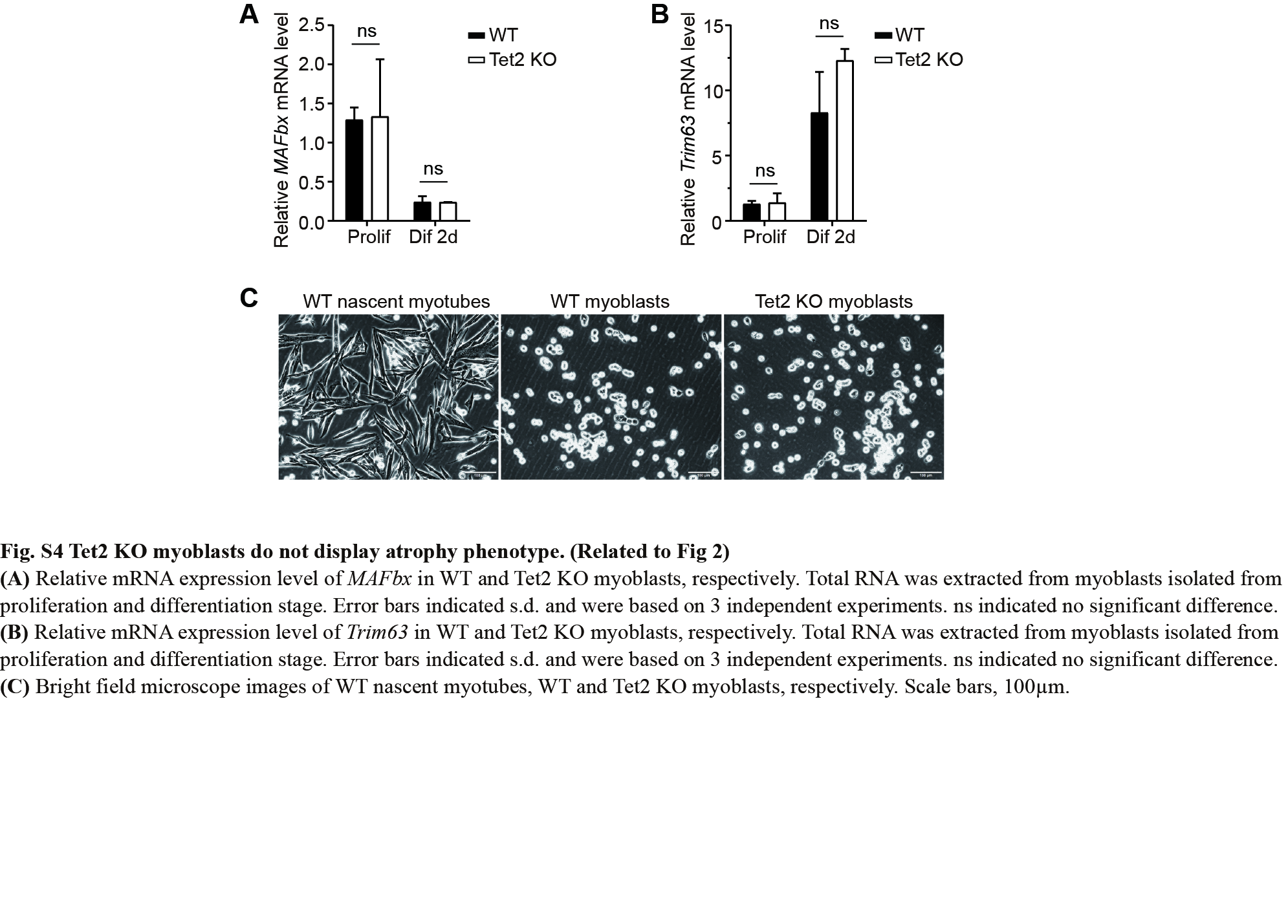

Supplement: Supplementary file 4 — Fig. S4 [file 41419_2021_3817_MOESM4_ESM.tif]

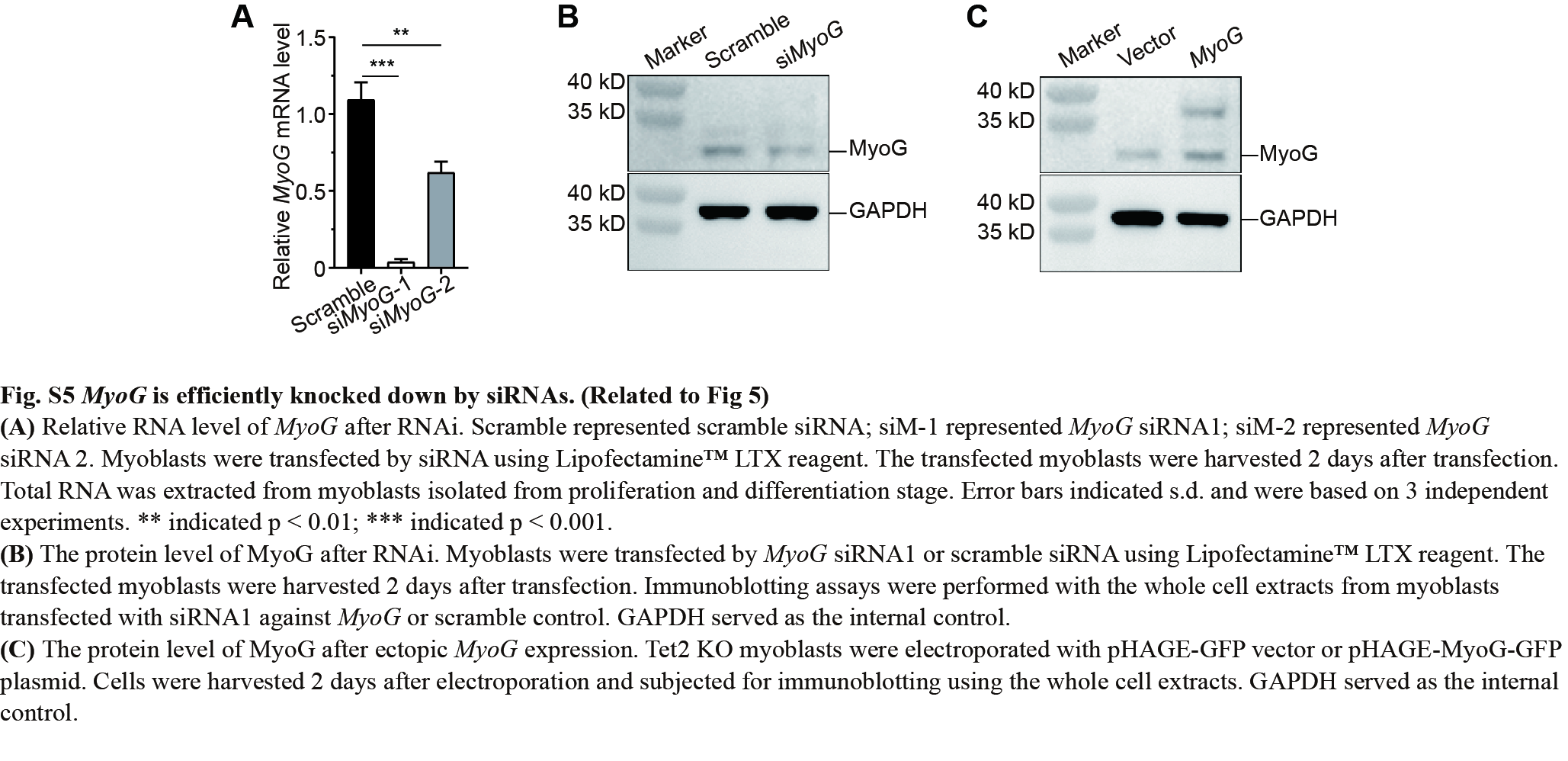

Supplement: Supplementary file 5 — Fig. S5 [file 41419_2021_3817_MOESM5_ESM.tif]

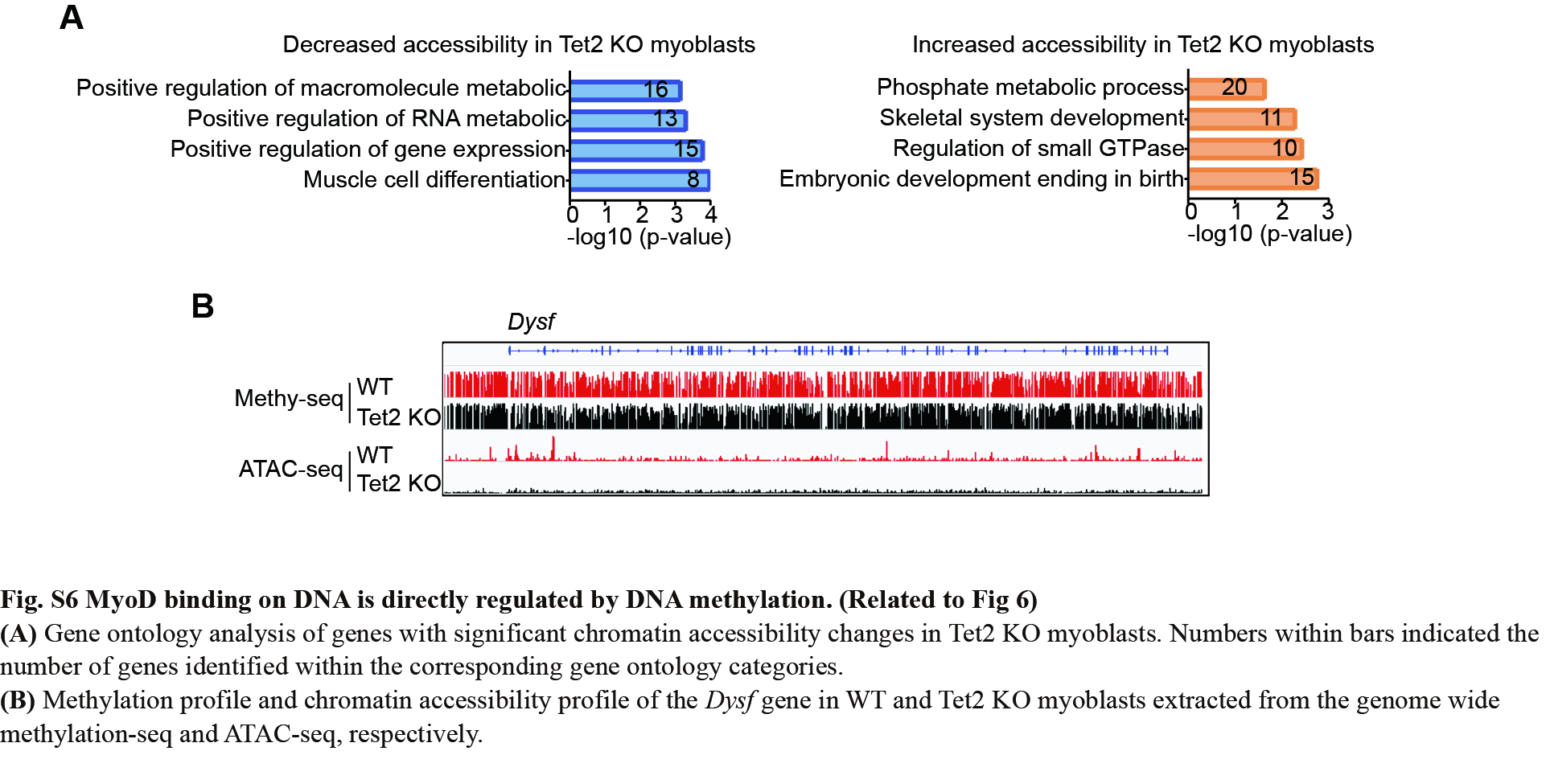

Supplement: Supplementary file 6 — Fig. S6 [file 41419_2021_3817_MOESM6_ESM.tif]

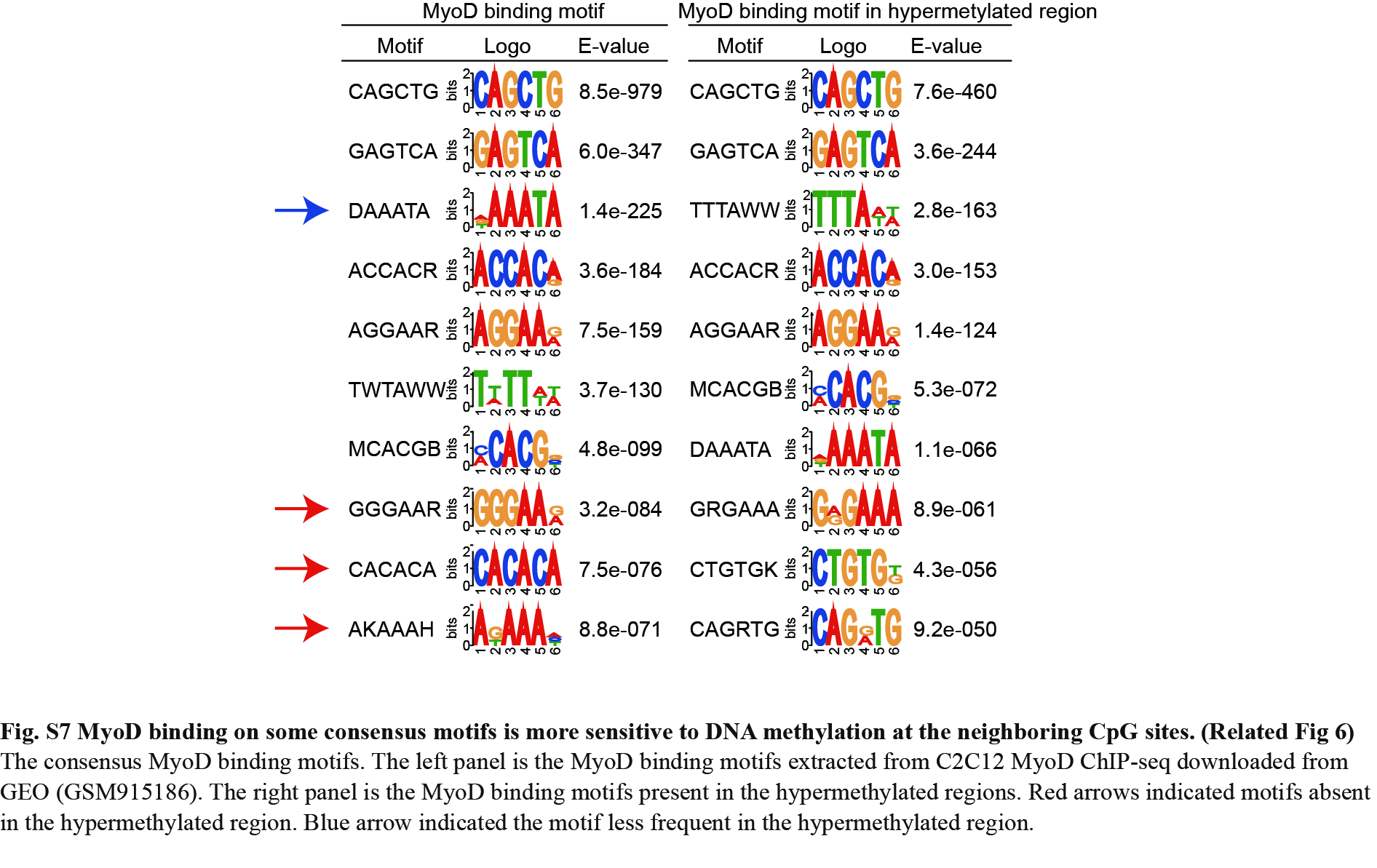

Supplement: Supplementary file 7 — Fig. S7 [file 41419_2021_3817_MOESM7_ESM.tif]
