# Supplementary material for: Muscle regeneration controlled by a designated DNA dioxygenase
Source: Cell Death Dis. 2021 May 25;12(6):535. doi: 10.1038/s41419-021-03817-2 (PMC8149877; doi:10.1038/s41419-021-03817-2)
Supplement: Supplementary file 8 — Table S1 [file 41419_2021_3817_MOESM8_ESM.docx]

**Table. S1 ChIP-qPCR primer list**

| Name | Sequence |
| --- | --- |
| Chip MyoD-F1 | GGAGATGGGGAGAGAGTGTG |
| Chip MyoD-R1 | TCCTCTCTGCCTCTGAAAGC |
| Chip MyoD-F2 | CAAGAGGCTTGGGGAAGAGA |
| Chip MyoD-R2 | CCCAGGTCCTCTTCAGTGAG |
| Chip-*MyoG* E2-F1 (F3) | CCAGCTTCCAATTCCCAACG |
| Chip-*MyoG* E2-R1 (R3) | GGTCATGAGAAAATCCCGTCC |
| Chip-*MyoG* E2-F2 (F4) | TGTTCCATGCCGTCCTTAGT |
| Chip-*MyoG* E2-R2 (R4) | CGTTGGGAATTGGAAGCTGG |
| Chip-*MyoG* E1-F1 (F5) | CTGTCCCTCCCACAGACACT |
| Chip-*MyoG* E1-R1 (R5) | ATTTTCTCACAGGCCAAACG |
| Chip-*MyoG* E1-F2 (F6) | TGCATTCCCAGAGTACACGA |
| Chip-*MyoG* E1-R2 (R6) | AGGGCACAGTAATGCCAGAT |
| Chip MyoD-F7 | CTGCAGAGGAGTAAGACGGA |
| Chip MyoD-R7 | GGCAGCAAGTAGGGTAGAGT |
| Chip MyoD-F8 | AAGATCCCATGGCTCCTCTC |
| Chip MyoD-R8 | AAGTGCGTGTGTTCTGGATG |
