# Supplementary material for: Muscle regeneration controlled by a designated DNA dioxygenase
Source: Cell Death Dis. 2021 May 25;12(6):535. doi: 10.1038/s41419-021-03817-2 (PMC8149877; doi:10.1038/s41419-021-03817-2)
Supplement: Supplementary file 9 — Table S2 [file 41419_2021_3817_MOESM9_ESM.docx]

**Table. S2 RT-qPCR primer list**

| Name | Sequence |
| --- | --- |
| *Tet1*-RT-F | ACAACATGCACAACGGAAGC |
| *Tet1*-RT-R | TTGGCCTTCATCCCTTCCAC |
| *Tet2*-RT-F | GTGCTGCTGGATTCATTCAAAG |
| *Tet2*-RT-R | GTATGGCTGATGGGAGAAGGTG |
| *Tet3*-RT-F | CTATCAGAACCAGGTGACCAATGAG |
| *Tet3*-RT-R | ACAGTGCACCCATTGTAGAGGTTAT |
| *Gapdh*-RT-F | GCCAGCCTCGTCCCGTAGACA |
| *Gapdh*-RT-R | CAACAATCTCCACTTTGCCACTGC |
| *Pax7*-RT-F | TTTCCCATGGTTGTGTCTCC |
| *Pax7*-RT-R | GTCGCAGTGACCGTCCTT |
| *Myf5*-RT-F | CCACCAACCCTAACCAGAGA |
| *Myf5*-RT-R | CTGTTCTTTCGGGACCAGAC |
| *MyoD*-RT-F | GCCTTCTACGCACCTGGAC |
| *MyoD*-RT-R | ACTCTTCCCTGGCCTGGACT |
| *MyoG*-RT-F | ACTCCCTTACGTCCATCGTG |
| *MyoG*-RT-R | CAGGACAGCCCCACTTAAAA |
| *Myh1*-RT-F | GACTACAACATCGCTGGCTG |
| *Myh1*-RT-R | CTTGGCCCCTTTCTTTCCAC |
| *Myh3*-RT-F | GAACTTGAAGGAGAGGTCGA |
| *Myh3*-RT-R | CACCTTCGCCTGTAATTTGTC |
| *Mck*-RT-F | CATGGAGAAGGGAGGCAATA |
| *Mck*-RT-R | GACGAAGGCGAGTGAGAATC |
| *Tmem8C*-RT-F | CCTGCTGTCTCTCCCAAG |
| *Tmem8C*-RT-R | AGAACCAGTGGGTCCCTAA |
| *Gm7325*-RT-F | GGACCACTCCCAGAGGAAGGA |
| *Gm7325*-RT-R | GGACCGACGCCTGGACTAAC |
| *Myom2*-RT-F | CTATGGGATTACCCTTCTCAACT |
| *Myom2*-RT-R | ACCGTTAAGATCCTCTCTTTGAC |
| *Dysf*-RT-F | GGGGGAGAAGATGAGTGACA |
| *Dysf*-RT-R | TTCTTGGCAACAGCACAGAC |
| *Mef2C*-RT-F | GCCAGTTACCATCCCAGTGT |
| *Mef2C*-RT-R | ATCAGACCGCCTGTGTTACC |
| *Casq2*-RT-F | ACCGTACACAGAAGAGGAGC |
| *Casq2*-RT-R | GGTCACTCTTCTCCGCAAAG |
| *Ccnb2*-RT-F | AGGTCTGTGAGGAACAAAAGTG |
| *Ccnb2*-RT-R | CACAGAGTTGTCGGTGTAAATG |
| *Cdk6*-RT-F | GGACAGAGAAACCAAGCTTACA |
| *Cdk6*-RT-R | TTCAGATCACGATGCACTACTC |
| *Rab32*-RT-F | GGAAGCTCTTGGAGCATTTG |
| *Rab32*-RT-R | TCCATCTGGGAAGGACTCTG |
| *Itgb2*-RT-F | GAAACTGTCGGAAGGACAATAG |
| *Itgb2*-RT-R | CGCAGACTTGGCTGTTATATCT |
| *Mapk13*-RT-F | GAGCTTCCATGATTTCTACCTG |
| *Mapk13*-RT-R | TCAGCTCACAGTCTTCATTCAC |
| *Tet2* (exon3)-RT-F1 | GTGCTGCTGGATTCATTCAAAG |
| *Tet2* (exon3)-RT-R1 | GTATGGCTGATGGGAGAAGGTG |
| *Tet2* (exon3)-RT-F2 | AGGGATAAAACGCACAGTCAGT |
| *Tet2* (exon3)-RT-R2 | TGCTTTCTCCCTTAGCTTTTTG |
| *Tet2* (exon3)-RT-F3 | TTGCACTCAGTCCTCCACTCT |
| *Tet2* (exon3)-RT-R3 | GACATTGCCAGTGGACTGTTT |
| *Tet2* (exon3)-RT-F4 | TGAGTCAACAGGCCACAGAG |
| *Tet2* (exon3)-RT-R4 | GTTCTGGGATGCTGGTGTTT |
| *Tet2* (exon3)-RT-F5 | GGGGTTGGAGCAAGTACAAA |
| *Tet2* (exon3)-RT-R5 | CGGGTGTGTGTCATTTGAAG |
