# Supplementary material for: Muscle regeneration controlled by a designated DNA dioxygenase
Source: Cell Death Dis. 2021 May 25;12(6):535. doi: 10.1038/s41419-021-03817-2 (PMC8149877; doi:10.1038/s41419-021-03817-2)
Supplement: Supplementary file 10 — Table S3 [file 41419_2021_3817_MOESM10_ESM.docx]

**Table. S3 Clone primer list**

| Name | Sequence |
| --- | --- |
| *MyoG*-promoter-F | CCGCTCGAGGGCCAAGATTCAATGGGAAT |
| *MyoG*-promoter-R | CCCAAGCTTTCACACCAACTGCTGGGTGC |
| *MyoG* E1-F | GGGGTACCGTTCCATTTTGGGAACTTAT |
| *MyoG* E1-R | CGACGCGTAAGGGCACAGTAATGCCAGAT |
| *MyoG* E2-F | GGGGTACCTGTGGTGTCAGGAAGTGTTC |
| *MyoG* E2-R | CGACGCGTGTTCCCAAAATGGAACCCTT |
| *MyoG* CDS -F | CTAGCTAGCATGGAGCTGTATGAGACATC |
| *MyoG* CDS-R | CCGCTCGAGGTTGGGCATGGTTTCGTCTG |
