# Supplementary material for: Muscle regeneration controlled by a designated DNA dioxygenase
Source: Cell Death Dis. 2021 May 25;12(6):535. doi: 10.1038/s41419-021-03817-2 (PMC8149877; doi:10.1038/s41419-021-03817-2)
Supplement: Supplementary file 11 — Table S4 [file 41419_2021_3817_MOESM11_ESM.docx]

**Table. S4 Point mutation primer list**

| Name | Sequence |
| --- | --- |
| *MyoG* E2 (1,2)-F | ATGCGGTCCTTAGTAGCTGTGAGATC |
| *MyoG* E2 (1,2)-R | GATCTCACAGCTACTAAGGACCGCAT |
| *MyoG* E2 (4,5)-F | CAAGGAGGCTTAATAATACCAGCTTCCAATTCCCAAGGTCAC |
| *MyoG* E2 (4,5)-R | GTGACCTTGGGAATTGGAAGCTGGTATTATTAAGCCTCCTTG |
| *MyoG* E2 (6,7)-F | TGGTGGTGAAGCTGGATGGGATTTTCTCAT |
| *MyoG* E2 (6,7)-R | ATGAGAAAATCCCATCCAGCTTCACCACCA |
