# Supplementary material for: Muscle regeneration controlled by a designated DNA dioxygenase
Source: Cell Death Dis. 2021 May 25;12(6):535. doi: 10.1038/s41419-021-03817-2 (PMC8149877; doi:10.1038/s41419-021-03817-2)
Supplement: Supplementary file 12 — Table S5 [file 41419_2021_3817_MOESM12_ESM.docx]

**Table. S5 Bisulfite-sequencing primer list**

| Name | Sequence |
| --- | --- |
| *MyoG* E1-bs-F | GTTTTATTTTGGGAATTTATTTAAG |
| *MyoG* E1-bs-R | AAAAACACAATAATACCAAATACC |
| *MyoG* E2-bs-F | TGTGGTGTTAGGAAGTGTTTTA |
| *MyoG* E2-bs-R | ATTCCCAAAATAAAACCCTTAA |
| *Mef2C*-1bs-F | TGGGTTTTTGTATAATTGTTAATT |
| *Mef2C*-1bs-R | AAATTAAAATCAATATTTCTCCAA |
| *Mef2C*-2bs-F | TTATTTTGTATTGGAGGTTGTATT |
| *Mef2C*-2bs-R | AATAAATTCTCACAAACCTTCC |
| *Myom2*-bs-F | GGGTAGGGAGTTTTTAGTTATTG |
| *Myom2*-bs-R | CAACCAACATAACTACAAACCA |
